# Percentage of Patients with Preventable Adverse Drug Reactions and Preventability of Adverse Drug Reactions – A Meta-Analysis

**DOI:** 10.1371/journal.pone.0033236

**Published:** 2012-03-15

**Authors:** Katja M. Hakkarainen, Khadidja Hedna, Max Petzold, Staffan Hägg

**Affiliations:** 1 Nordic School of Public Health (NHV), Gothenburg, Sweden; 2 Laboratoire d'Enseignement et de Recherche sur le Traitement de l'Information Médicale, Université de la Méditerrané, Marseille, France; 3 Centre for Applied Biostatistics, University of Gothenburg, Gothenburg, Sweden; 4 Department of Drug Research/Clinical Pharmacology, Linköping University, Linköping, Sweden; University of Michigan, United States of America

## Abstract

**Background:**

Numerous observational studies suggest that preventable adverse drug reactions are a significant burden in healthcare, but no meta-analysis using a standardised definition for adverse drug reactions exists. The aim of the study was to estimate the percentage of patients with preventable adverse drug reactions and the preventability of adverse drug reactions in adult outpatients and inpatients.

**Methods:**

Studies were identified through searching Cochrane, CINAHL, EMBASE, IPA, Medline, PsycINFO and Web of Science in September 2010, and by hand searching the reference lists of identified papers. Original peer-reviewed research articles in English that defined adverse drug reactions according to WHO’s or similar definition and assessed preventability were included. Disease or treatment specific studies were excluded. Meta-analysis on the percentage of patients with preventable adverse drug reactions and the preventability of adverse drug reactions was conducted.

**Results:**

Data were analysed from 16 original studies on outpatients with 48797 emergency visits or hospital admissions and from 8 studies involving 24128 inpatients. No studies in primary care were identified. Among adult outpatients, 2.0% (95% confidence interval (CI): 1.2–3.2%) had preventable adverse drug reactions and 52% (95% CI: 42–62%) of adverse drug reactions were preventable. Among inpatients, 1.6% (95% CI: 0.1–51%) had preventable adverse drug reactions and 45% (95% CI: 33–58%) of adverse drug reactions were preventable.

**Conclusions:**

This meta-analysis corroborates that preventable adverse drug reactions are a significant burden to healthcare among adult outpatients. Among both outpatients and inpatients, approximately half of adverse drug reactions are preventable, demonstrating that further evidence on prevention strategies is required. The percentage of patients with preventable adverse drug reactions among inpatients and in primary care is largely unknown and should be investigated in future research.

## Introduction

Drug-related adverse events, including adverse drug reactions (ADRs), have been reported to be among leading causes of morbidity and mortality [1], [2]. ADRs occur in both outpatients and inpatients [2]-[7]. In a meta-analysis in 2002, 4.9% of hospital admissions were associated with ADRs, ranging between 0.2 and 41.3% in individual studies [4]. Further, 28.9% of the ADR-related hospitalisations were considered preventable. Of inpatients, 10.9% is estimated to experience an ADR during hospitalisation [2]. According to the World Health Organization (WHO), costs of ADRs, including hospitalisations, surgery and lost productivity, exceed the cost of medicines in some countries [8]. As drug-related adverse events are estimated to cost USD 422–7062 per drug-related admission and USD 2284–5640 per inpatient with drug-related adverse events (2000 values) [9], significant costs may be saved if drug-related adverse events, including ADRs, were prevented.

Previous review studies have investigated preventable drug-related adverse events and the preventability of the events [4], [6], [10]-[13]. However, most of them summarised studies on all drug-related adverse events, including adverse events due to noncompliance and overdose, without a clear definition for the adverse event, and used medians as summary measures [6], [11]-[13]. Two previous reviews investigated the preventability of ADRs among outpatients being admitted to hospital [4], [10], but no standardised definition for ADRs was required, outpatients without hospitalisation were not studied and no meta-analysis technique was used to pool the results. Further, no previous review has investigated the preventability of ADRs among inpatients or the percentage of outpatients or inpatients with preventable ADRs (PADRs). Therefore, we applied meta-analysis techniques using a standardised definition for ADRs to estimate the percentage of adult outpatients and inpatients with PADRs, and the preventability of ADRs.

## Methods

We carried out a meta-analysis on studies on PADRs in adults, adapting methods recommended by the Statement for Reporting Systematic Reviews and Meta-analyses [14], and following our study protocol. We searched seven databases; MEDLINE, Excerpta Medica Database (EMBASE), the Cochrane database of systematic reviews, Cumulative Index to Nursing & Allied Health Literature (CINAHL), International Pharmaceutical Abstract (IPA), PsycINFO and Web of Science (–September 2010) for relevant publications. References of included articles and previous reviews and meta-analysis on ADRs were reviewed to identify additional relevant articles and consider their inclusion.

**Figure 1:**
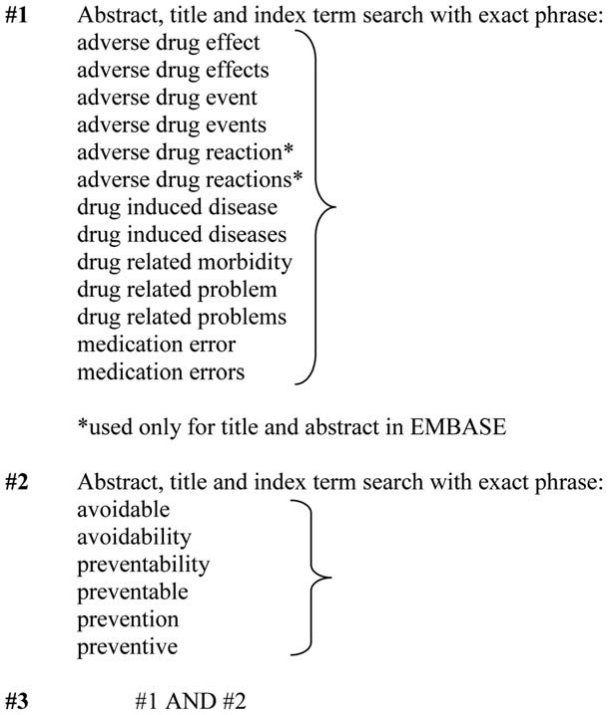
Search strategy used in the search of eight bibliographic databases.

The databases’ search fields for titles, abstracts and index terms were searched using the databases’ index terms and other commonly utilised terminology on drug-related adverse events and preventability (Figure 1). No limits for the years of publication were set. The search was limited to English. Multiple publications of the same study were carefully reviewed [15]. The titles and abstracts were screened by one researcher (KMH). Studies were selected for inclusion from full-text articles in collaboration by two researchers (KH, KMH). An additional reviewer (SH) participated in the review process when uncertainty about eligibility criteria arose.

We included original peer-reviewed research articles published in English regardless of the study design (prospective, retrospective, cross-sectional, or interventional). To avoid inconsistent estimates, ADRs had to be defined according to the WHO: “*a response which is noxious and unintended, and which occurs at doses normally used in humans for the prophylaxis, diagnosis, or therapy of disease, or for the modification of physiological function*” [16], or according to Edwards and Aronson’s similar definition [17]. Small changes in wording were overlooked if the two researchers (KH, KMH) agreed that the functional meaning remained the same. Studies representing one or two specific disease areas (inclusion of patients, setting or sampling frame) or specific treatments were excluded. Included studies had to report the percentage of patients with PADRs or the preventability of ADRs. We excluded articles summarising previous results without original assessment of ADRs and the preventability of ADRs and studies conducted in paediatrics or intensive care, or focusing only on specific types of, life threatening or fatal ADRs. We also excluded studies if ADRs were identified exclusively through spontaneous reporting or International Classification Disease (ICD-9 or 10) codes, as these two strategies are known to underestimate the rate of ADRs [7], [18]. Although we set no limitations on how preventability must be defined in original studies, we required a case by case preventability assessment. Thus, we excluded studies that considered all type A ADRs, defined as dose dependent and predictable from the known pharmacological characteristics of the drug [19], as preventable without a separate preventability assessment. Studies were also excluded if the percentage of patients with PADRs and preventability of ADRs were inadequately reported.

### Data extraction

To increase the reliability and efficiency of data extraction, we developed and piloted a paper data extraction form by adapting the checklist for Strengthening the Reporting of Observational Studies in Epidemiology [20]. Studies’ characteristics such as the study design, data source, sampling frame, population characteristics, and definition for ADRs, as well as the number of included patients, healthcare visits, ADRs, PADRs, patients with PADRs, and healthcare visits with PADRs were extracted by two researchers. The first (KH) extracted the data, and the second (KMH) confirmed the accuracy and completeness of the extraction. Any disagreements were noted and resolved by consensus. The extracted data were based on information reported in or calculated from the included articles. Authors were not contacted for complementary information.

The number of PADRs in each study represented the sum of definitely, probably or possibly preventable cases, as reported in original studies. The percentage of patients with PADRs was calculated by dividing the reported number of healthcare visits (such as primary or emergency care visits or hospitalisations) with PADRs by the total number of healthcare visits. The percentages could be calculated only if the number of healthcare visits were interpretable in the original studies. The preventability of ADRs was calculated by dividing the number of PADRs by the total number of ADRs. If the number of PADRs, ADRs, healthcare visits or healthcare visits with PADRs was not directly reported, the two reviewers assessed whether they were interpretable based on other presented data.

### Risk of bias

The two reviewers (KH, KMH) assessed independently the quality and the risk of bias in the original studies in conjunction with data extraction. To minimise inconsistent estimates, we required a standardised definition for ADRs [16], [17], a case by case assessment of preventability, and more inclusive data sources than spontaneous reports or International Classification Disease (ICD-9 or 10) codes exclusively. No scoring system for quality assessment of the individual studies was applied, as no consensus on quality scoring of observational epidemiological studies exists [21].

### Statistical analysis

Meta-analysis was performed using DerSimonian and Laird random effect model with the estimate of heterogeneity being taken from the inverse variance fixed effect model [22]. The summary measures for the percentage of patients with PADRs and for the preventability of ADRs were calculated separately for ADRs occurring in outpatients and for ADRs present among inpatients during hospitalisation. Studies on the elderly were analysed separately. For the percentage of patients with PADRs, we first converted the individual percentage estimates to logit to meet the normal distribution assumption, conducted the analysis on the logit and converted the final results into non-logit for interpretation. Preventability estimates were calculated without converting them into logit in the analysis, because overall estimates using direct and logit methods differed less than one percentage when they were compared. Confidence intervals (95%) for each summary measure were calculated. STATA software version 10 was used for data analysis.

To investigate the robustness of the overall estimates, we conducted sensitivity analyses. Each analysis was conducted separately for studies with less than six months’ study period and with six months’ or longer study period, because studies with longer study periods may more likely include revisits of the same patients. Moreover, outlier studies whose estimates differed 20% or more from the overall estimate were omitted from each analysis. We also assessed the possibility of publication bias by evaluating funnel plots. No asymmetry was evident. Heterogeneity was explored using Cochrane’s Q test of heterogeneity and I^2^ statistics.

### Ethics Statement

According to Swedish regulations on medical research on humans, approval by an ethical review board is not required in review studies and meta-analyses that use aggregated patient data from previous studies. As no individual patient data was used or stored for the current study, informed consents from the participants of the original studies were not required.

## Results

**Figure 2:**
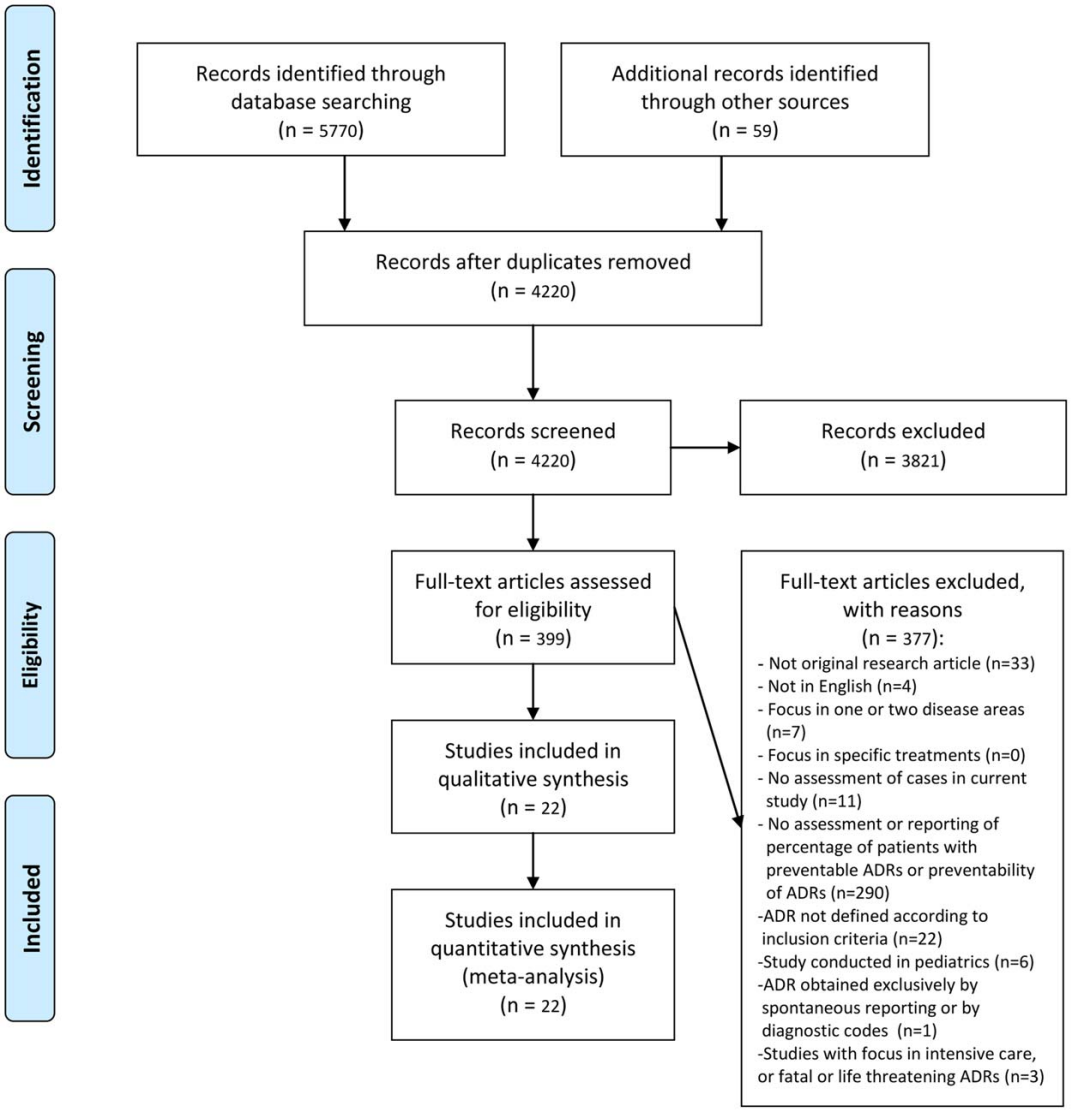
Flow diagram of the selection of eligible studies on preventable adverse drug reactions (ADR).

A total of 5770 citations were found from electronic database searches and additional 59 records were identified from reference lists (Figure 2). After removal of duplicate records, the inclusion and exclusion criteria were applied on 4220 unique citations. After title and abstract review, the inclusion and exclusion criteria were applied on 399 articles’ full texts. Most full-text articles were excluded due to not assessing or reporting the percentage of patients with PADRs or the preventability of ADRs (n = 290). Excluded articles commonly focused on potential drug-related problems or other types of adverse events without a category for ADRs or their preventability. Many also lacked a denominator for calculating the percentage of patients or preventability. After applying all inclusion criteria, we finally included 22 articles in the review. Of these, 14 studies reported PADRs among outpatients exclusively, six among inpatients, and two separately for outpatients and inpatients (Tables 1,2,3).

**Table 1:** Characteristics of included studies on preventable adverse drug reactions among outpatients being hospitalisedφ.

Study (Country)	Study period	Study design	Population characteristics	Characteristics of patients with ADR	Definition for ADR‡	Criteria for preventability
**Pearson 1994** [23] (United States)†	6 months, 1992–1993	Prospective, observational	No age limitation	Mean age unknown, 50% male	Similar to WHO [16]	Schumock [40]
**Courtman 1995** [24] (Canada)	20 weeks, 1992–1993	Prospective, observational	Age>65 years, median 78 years (range 65–108), 41% male	Unknown	WHO [16]	Modified Hallas [39]
**Dartnell 1999** [25] (Australia)	30 days, 1994	Prospective, observational	Median age 58 years (range 15–91), 38% male	Unknown	WHO [16]	Own criteria
**Chan 2001** [27] (Australia)	8 weeks, 1998	Cross-sectional, observational	Mean age 82 years (range 75–94), 45% male	Unknown	WHO [16]	Modified Hallas [39]
**Olivier 2002** [28] (France)	Weeks, 1998	Prospective, observational	Age>15 years	Mean age 58 years, 54% male	WHO [16]	Imbs [57] and own criteria#
**Dormann 2003** [29] (Germany)	13 months, 1998–1999	Prospective, observational	Mean age 54 years (range 17–97), 85% male	Unknown	WHO [16]	Schumock [40]
**Dormann 2004** [30] (Germany)†	12 months+6 months for related readmission, no year	Prospective, observational	Mean age 57 years (range 18–97), high proportion of those between 55–70 years	Unknown	WHO [16]	Schumock [40]
**Pirmohamed 2004** [31] (United Kingdom)	6 months, 2000–2002	Prospective, observational	Median age 66 years (range 46–79), 48% male	Median age 76 years (range 65–83), 41% male	Edwards and Aronson [17]	Hallas [39]
**Alexopoulou 2008** [33] (Greece)	6 months, 2005	Prospective observational	Mean age 65 years (range 15–100), 2/3 participants>65 years, 50% male	Mean age 71 years (range 69–73), 47% male	WHO [16]	Modified Hallas [39]
**Fransceschi 2008** [34] (Italy)	14 months, 2004–2005	Prospective, observational	Age ≥65 years	Mean age 77 years (range 60–93), 41% male	Edwards and Aronson [17]	Hallas [39] integrated with Gurwitz [58]
**Hopf 2008** [35] (United Kingdom)	2 weeks, 2006	Prospective, observational	People without ADR: mean age 62 years, 35% male	Mean age 67years (range 19–91), 43% male	WHO [16]	Hallas [39]
**Ruiz 2008** [36] (Spain)*	21 months, 2001–2003, for each patient 60 days	Prospective, observational	Unknown	Aged>65 years represent 65%, 58% male	WHO [16]	Schumock [40]
**Van Der Hooft** **2008** [37] (Netherlands)	12 months, 2003	Retrospective, observational	Mean age 38 years, 48.5% male	Unknown	Similar to WHO [16]	Hallas [39]

ADR = adverse drug reaction; WHO = World Health Organization.

φ ADRs are present at admission and may or may not be the main reason for hospitalisation.

† Data from study used for analyses for both outpatients and inpatients.

*ADR reason for re-hospitalisation.

‡ Studies using WHO’s definition may have referenced another publication for the definition.

# Compared the two methods for assessing preventability of which the “own criteria” was chosen to be included in the meta-analysis.

### Study characteristics

Sixteen included studies reported PADRs among outpatients being hospitalised or visiting emergency care (Tables 1,2) [23]-[38]. We did not identify any studies in primary care without a hospitalisation or emergency visit. Nine studies were conducted in Europe [28]-[31], [33]-[37], two in Australia [25], [27], four in North America [23], [24], [26], [38], and one India [32]. The studies were conducted between 1992 and 2006. The study periods ranged from two weeks [35] to 21 months [36]. Fourteen studies had a prospective design [23]-[26], [28]-[36], [38], one retrospective [37], and one cross-sectional [27]. All 16 studies used medical records as the source of information, combined to reporting of ADRs or interviewing patients or their family. Of these, 11 studies were conducted in some wards [24]-[30], [32]-[34], [38], four included the whole hospital [23], [31], [35], [36], and one used a population database of general practitioners as a source of recruitment [37]. Three studies were conducted exclusively in the elderly (≥65 years) [24], [27], [34]. Except for two studies [24], [32], all assessed the preventability by the consensus of at least two professionals [23], [25]-[31], [33]-[38]. For determining preventability, the criteria introduced by Hallas [39] were used in nine of the 16 studies [24], [27], [31]-[35], [37], [38], and Schumock’s criteria [40] in four [23], [29], [30], [36].

**Table 2:** Characteristics of included studies on preventable adverse drug reactions among outpatients visiting emergency care.

Study (Country)	Study period	Study design	Population characteristics	Characteristics of patients with ADR	Definition for ADR‡	Criteria for preventability
**Patel 2007** [32] (India)	6 weeks, 2005	Prospective, observational	Age>18 years	Mean age 40 years.	WHO [16]	Modified Hallas [39]
**Zed 2008** [38] (Canada)	12 weeks, 2006	Prospective, observational	Mean age 50 years, 48% male	Unknown	Similar to WHO [16]	Hallas [39]
**Tafreshi 1999** [26] (United States)	35 days, 1996	Prospective, observational	Mean age 53 years (range 0, 1–95), 43% male	Unknown	Similar to WHO [16]	Own criteria

ADR = adverse drug reaction; WHO = World Health Organization.

‡ Studies using WHO’s definition may have referenced another publication for the definition.

**Table 3:** Characteristics of included studies on preventable adverse drug reactions among inpatients.

Study (Country)	Study period	Study design	Population characteristics	Characteristics of patients with ADRs	Definition for ADR‡	Criteria for preventability
**Pearson 1994** [23] (United States)†	6 months, 1992–1993	Prospective, observational	No age limitation	Mean age unknown, 50% male	Similar to WHO [16]	Schumock [40]
**Gholami 1999** [41] (Iran)	4 months, 1996	Prospective, randomised for inclusion	No age limitation, range 10–86 years	Unknown	WHO [16]	Schumock [40]
**Dormann 2004** [30] (Germany)†	12 months+6 months for related readmission, no year	Prospective, observational	Mean age 57 years (range 18–97), high proportion of those between 55–70 years	Unknown	WHO [16]	Schumock [40]
**Baniasadi 2008** [42] (Iran)	12 months, 2006–2007	Prospective, interventional	No age limitation	Unknown	WHO [16]	Schumock [40]
**Davies 2006** [43] (United Kingdom)	2 weeks, 2005	Prospective, observational	Median age 61 years (range 45–78), 51% male	Median age 70 years (range 52–79), 38% male	Edwards and Aronson [17]	Hallas [39]
**Davies 2009** [44] (United Kingdom)	6 months, 2005	Prospective, observational	Median age of people without ADR 61 years	Median age 72 years, 41% male	Edwards and Aronson [17]	Hallas [39]
**Pourseyed 2009** [45] (Iran)	15 weeks, 2004	Prospective, observational	Mean age 60 years (range 13–91), 78% between 40–79 years, 51% male	Mean age 54 years, 43% male	WHO [16]	Schumock [40]
**Farcas 2010** [46] (Romania)	12 months, 2009	Prospective, observational	Mean age 59 years (range 25–92), 47% male	Mean age 65 years, 31% male	WHO [16]	Imbs [57]

ADR = adverse drug reaction; WHO = World Health Organization.

† Data from study used for analyses on preventable adverse drug reactions for both outpatients and inpatients.

‡ Studies using WHO’s definition may have referenced another publication for the definition.

Eight included studies conducted between 1992 and 2009 investigated inpatients’ PADRs that were present during hospitalisation (Table 3) [23], [30], [41]-[46]. All studies had a prospective design. Four studies were conducted in Europe [30], [43], [44], [46], one in North America [23] and three in Iran [41], [42], [45]. The study periods ranged from two weeks [43] to 18 months [44]. Except for one study [46], all used medical records as the source of information, combined to reporting of ADRs or interviewing patients or their family. Five studies were conducted in some wards [30], [43], [44], [46], and three included the whole hospital [23], [41], [42]. All studies included adults of all ages. In six studies [23], [30], [43]-[46], preventability was assessed by the consensus of at least two professionals, and the number of assessors was unclear in two [41], [42]. All studies [23], [30], [41]-[45], except for one [46], used either Hallas [39] or Schumock [40] criteria for determining preventability.

### Preventable adverse drug reactions among outpatients

**Figure 3:**
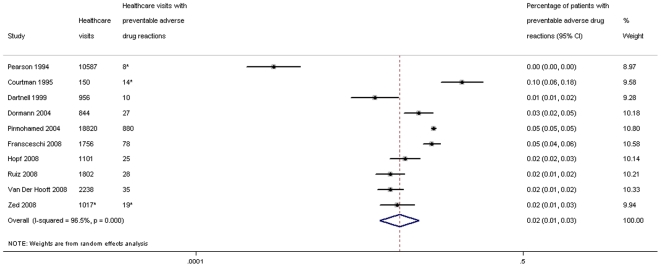
Percentage of patients with preventable adverse drug reactions among outpatients being hospitalised or visiting emergency care. *not provided directly in the study, interpreted from other presented data.

**Figure 4:**
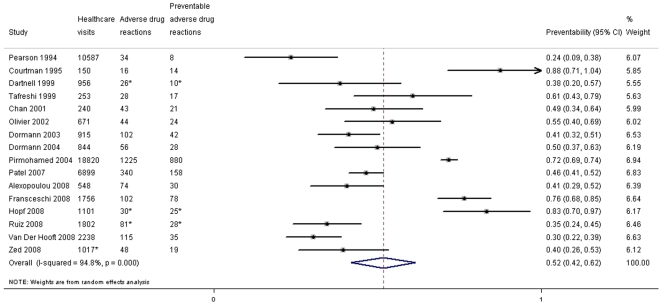
Preventability of adverse drug reactions among outpatients being hospitalised or visiting emergency care. *not provided directly in the study, interpreted from other presented data.

The 16 studies on outpatients involved 48797 emergency visits or hospital admissions. PADRs occurred in 2% (95% confidence interval (CI): 1.2–3.2%) of outpatients (Figure 3), and 52% (95% CI: 42–62%) of ADRs present at the time of hospitalisation or an emergency visit were preventable (Figure 4). The preventability was higher in the three studies including only the elderly, for which 71% (95% CI: 51–91%) of ADRs were preventable.

### Preventable adverse drug reactions among inpatients

**Figure 5:**
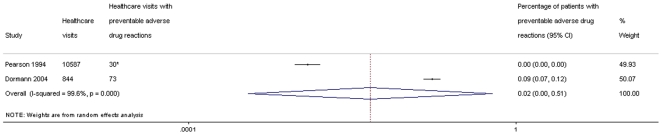
Percentage of inpatients with preventable adverse drug reactions, during hospitalisation. *not provided directly in the study, interpreted from other presented data.

**Figure 6:**
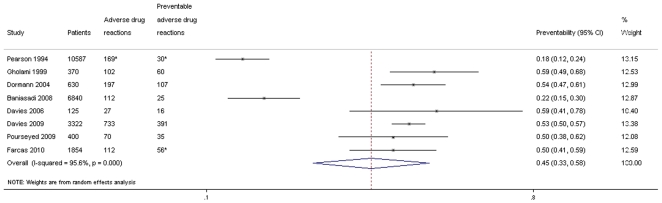
Preventability of adverse drug reactions among inpatients, during hospitalisation. *not provided directly in the study, interpreted from other presented data.

In total 24128 inpatients were included in the eight studies on ADRs present during hospital stay. In inpatients, 1.6% (95% CI: 0.1–51%) experienced a PADR during hospital stay (Figure 5). Among inpatients, we found that 45% (95% CI: 33–58%) of ADRs were assessed as preventable (Figure 6).

### Sensitivity analyses

The preventability of ADRs among outpatients and inpatients and the percentage of outpatients with PADRs were higher in studies with shorter study periods. When outliers were removed from the main analysis, the preventability of ADRs among outpatients was 45% (95% CI: 40–59%), lower than in the main analysis. The preventability of ADRs among inpatients was 54% (95% CI: 51–56%), higher than in the main analysis, when outliers were removed. However, all sensitivity analyses’ estimates, both according to study period and after removal of outliers, were within the confidence intervals of the overall estimates of the main analyses.

## Discussion

We found that 2% of adult outpatients being hospitalised or visiting emergency care experience PADRs. Approximately half of all ADRs among outpatients were preventable. As no studies in primary care were identified, the percentage of patients with PADRs and the preventability of ADRs remain unknown among outpatients without an admission or emergency visit. Among inpatients, close to half of ADRs present during hospitalisation were preventable, but the percentage of inpatients with PADRs could not be estimated precisely.

### Strengths and limitations of the review

Our study is the first to estimate in a meta-analysis the percentage of patients with PADRs and the preventability of ADRs, among both outpatients and inpatients. Our search strategy was comprehensive, but laborious with a large number of citations. We included published studies in English, as research is to be shared internationally, but this may have lead to overlooking some relevant studies. Authors were not contacted for unpublished data which may have lead to excluding studies that would have fulfilled the inclusion criteria if more data had been available. Further, although an excluded full-text article could include several exclusion criteria, only one exclusion criterion per article was recorded in the order of notifying the criterion. As it was not interpretable in most original studies whether ADRs were incident or prevalent, we used the percentage of patients with PADRs as an outcome measure. To prevent heterogeneous estimates and avoid bias, we required a standardised definition for ADRs, inclusive data sources and an original preventability assessment. Yet, the included studies were heterogeneous, perhaps due to varying study designs, settings and criteria for preventability. However, our overall estimates did not differ substantially when two sensitivity analyses were performed.

We found ADRs among adult outpatients more preventable than in earlier reviews, in which the median and pooled preventability of ADRs among patients being hospitalised has been 31% and 29%, respectively [4], [10]. The difference may arise from inconsistent definitions for ADRs. For providing consistent estimates, we required WHO’s [16] or a similar [17] definition for ADRs. Studies included in previous reviews on outpatients accepted various definitions for ADRs [4], [10], some of which found ADRs less preventable [47]-[50]. The criteria for preventability may also have influenced differing preventability estimates. While it was not an inclusion criterion, most studies in our meta-analysis used the criteria introduced by Hallas et al [39] or Schumock et al [40] to establish preventability. Some studies in previous reviews used more narrow criteria, considering exclusively inappropriate drug selection or dose [47] and unnecessary therapy [51] preventable. Thus, preventability due to other reasons, such as lack of monitoring or prescribing a prophylactic medication for an expected ADR, may have been overlooked in previous studies resulting in lower preventability estimates. In addition, 12 out of 16 articles on outpatients in our meta-analysis were published in the 21^st^ century while all articles in the previous reviews on PADRs were published in the 20^th^ century. Even though it may not be concluded from our results that the preventability of ADRs among outpatients would have increased over time, the increasing interest in and discussion on patient safety since the late 1990s’ [52] may have fostered acknowledging preventability in newer studies.

The lack of other review studies on the percentage of outpatients with PADRs or the preventability of ADRs among inpatients hinders comparison to previous evidence. In one review on all drug-related adverse events, at median 4.3% of all admissions among outpatients were considered drug-related and preventable [11]. Their higher estimate compared to ours on PADRs exclusively (2%) was expected, as they included events beyond ADRs, such as therapeutic failures, drug intoxications and misuse. Among inpatients, previous reviews have found that the median preventability of all drug-related adverse events is 35% and 46% [6], [12], ranging between 19% and 90% in individual studies. These are comparable to our estimate that 45% of ADRs are preventable among inpatients experiencing ADRs during hospital stay. Compared to previous reviews, our meta-analysis provides more consistent estimates on the preventability of ADRs and the proportion of patients with PADRs as our outcome measure is more standardised.

### Study implications

This meta-analysis demonstrates that PADRs are a significant cause of morbidity among outpatients and that roughly half of all ADRs among adult outpatients and inpatients may be prevented. In the included articles resulting in these preventability estimates, a common criteria for preventability was “*the drug event was due to a drug treatment procedure inconsistent with present-day knowledge of good medical practice or was clearly unrealistic, taking the known circumstances into account*” [39]. Others considered ADRs preventable when they occurred due to contraindications, inappropriate dose or monitoring, interactions, ignoring toxic serum drug concentrations or previous allergic reactions, or noncompliance [40]. ADRs occurring for these reasons need to be diminished to reduce the burden of ADRs, related costs [31], [44], and unnecessary patient harm. Thus, effective intervention strategies and safety measures for preventing ADRs need to be incorporated into healthcare in system-level. As support for prevention strategies, such as medication reviews, to reduce medication-related harm is limited [53], further evidence on interventions to prevent ADRs and their implementation in healthcare is required. However, errors related to use of medications will to some extent always occur mainly due to the human imperfection in mental functioning and due to the complex nature of medical practice [54].

Our meta-analysis also highlights the lack of evidence on PADRs. Despite our thorough search strategy, we did not identify studies on PADRs occurring in primary care. Thus, our findings are likely to represent only the most serious PADRs among outpatients. Further, only two studies allowed estimating the percentage of inpatients with PADR, and the generated overall estimate was imprecise. Therefore, future research should investigate PADRs in the general population, especially among people without emergency visits or hospitalisation and among inpatients during hospital stay. As identified in previously [55], [56], better consensus on defining and assessing preventability should also be reached to decrease heterogeneity between studies and enable more precise estimates in future meta-analyses.

This meta-analysis corroborates that PADRs are a significant burden to healthcare among adult outpatients. Among both outpatients and inpatients, approximately half of all ADRs are preventable. Although preventability estimates vary across studies, our results demonstrate that further evidence on prevention strategies is required. The percentage of patients with PADRs among inpatients and in primary care is largely unknown and should be investigated in future research.

## Supporting Information

Checklist S1 (DOC)

Protocol S1 (DOC)
