# Supplementary material for: Percentage of Patients with Preventable Adverse Drug Reactions and Preventability of Adverse Drug Reactions – A Meta-Analysis
Source: PLoS One. 2012 Mar 15;7(3):e33236. doi: 10.1371/journal.pone.0033236 (PMC3305295; doi:10.1371/journal.pone.0033236)
Supplement: Protocol S1 — (DOC) [file pone.0033236.s002.doc]

**Percentage of patients with preventable adverse drug reactions and preventability of adverse drug reactions – a meta-analysis**

**Study Protocol**

# Introduction

Medicines are normally effective and safe as treatments for the majority of diseases and medication therapy is the most common therapy in health care. However, adverse drug reactions are among the leading causes of morbidity and mortality in many countries (1, 2). Furthermore, it results in increased health care utilisation and excessive costs (3). According to WHO, ADR-related costs, such as hospitalisations, surgery and lost productivity, exceed the cost of the medications in some countries (4).

ADRs occur both in outpatients and inpatients (5, 6, 7) causing hospital admissions and prolongation of hospital stay (5). A large proportion of ADRs occurs as a result of a medication error, a failure in the medication use process, and is thus preventable. In order to introduce interventions for reducing drug-related morbidity and associated costs, more evidence is required on its prevalence and preventability in the general population and related costs to society and individuals.

We are not aware of any systematic review study that has estimates the percentage of patients with PADRs and the preventability of ADRs in both inpatient and outpatient settings. Therefore, this review is undertaken for this purpose, following the principles of the Preferred Reporting Items for Systematic Reviews and Meta-Analysis: The PRISMA statement (8).

# Objectives

- Determine the percentage of patients with preventable adverse drug reactions (PADRs) in inpatient and outpatient settings.
- Determine the preventability of adverse drug reactions (ADR) in inpatient and outpatient settings.

# Methods

## Eligibility criteria

### Studies design

Only original peer-reviewed research articles published in English will be included in the review, regardless of the design (observational, interventional, retrospective, prospective, or cross-sectional study). Both inpatient and outpatient setting studies will be included.

Articles will be excluded if they:

- are commentaries, editorials, letters, guidelines, reports, conference proceedings, case reports, and conceptual papers.
- represent one or two specific disease areas (setting, sampling frame), or specific treatments.
- summarise previous results without original assessment of ADRs and the preventability of ADRs.
- focus only on specific types of, life threatening or fatal ADRs.

### Participants

Articles will be included in the review regardless of the gender and age distribution of the study participants. However, studies on exclusively paediatric patients will be excluded. Articles in which study participants receive a specific treatment or have a specific health conditions will be excluded.

## Outcomes

- The percentage of patients with preventable ADRs, as incidence and prevalence are rarely available in original studies.
- The preventability of ADRs.

These outcome measures have to be reported or interpretable in the results sections of the studies. Articles summarising previous results without original assessment of ADRs and the preventability of ADRs will be excluded from the review. Articles will be excluded if outcome measures represent pharmacodynamic, pharmacokinetic and pharmacogenetic measures or chemical compounds other than medicines, or specific sub-types of ADRs rather than ADRs in general, e.g. ADRs exclusively due to interactions.

## Information sources

Search databases including the Cochrane database of systematic reviews, Cumulative Index to Nursing & Allied Health Literature (CINAHL), Excerpta Medica Database (EMBASE), International Pharmaceutical Abstract (IPA), MEDLINE, PsycINFO and Web of Science (–September 2010) will be searched by one researcher (KMH). The search will be limited to articles published in English. No limits for the years of publication will be set. References of included original articles and relevant review articles identified in the database search will be also retrieved.

## Search

Databases’ index terms and other commonly utilised terminology on ADRs and preventability will be used as search terms (Appendix 1).

## Study Selection

First, inclusion and exclusion criteria will be applied on the titles of citations found in the searched databases and on those identified from reference lists of included studies and from relevant reviews. Multiple records of the same citation from different databases will be removed and considered as a single citation. Subsequently, abstracts will be reviewed against the same criteria. Titles and abstracts will be screened by one researcher (KMH). Based on the abstract review, and if a definite decision can not be made based on the title/abstract, the inclusion and exclusion criteria will be applied on the remaining articles’ full texts. The screening and selection of full-text articles will be done in collaboration by two researchers (KMH, KH). The research group will be consulted, when required. Inclusion decision for each article will be recorded in a paper format indicating the reason for each article’s inclusion/exclusion.

## Data collection process

A paper data extraction form will be piloted on a sample of 10 included articles to ensure that all relevant information is captured (Appendix 2). When the items of the paper form have been tested, they will be entered in an Excel data base. Two researchers will perform the data extraction (KH, KMH). One will first extract the data (KH), and a second will independently check the data extraction forms for accuracy and completeness (KMH). Any disagreement will be noted and resolved by consensus between the two reviewers. If no agreement can be reached, a third reviewer (SH) would decide. The extracted data will be based on information reported in the included articles. Authors will not be contacted when information is missing.

Multiple publications of the same study will be carefully reviewed to avoid double recording of study participants which can lead to a biased result in a meta-analysis. Suspected multiple reporting will be scrutinised by comparing the country, sampling frame, study period, settings, participants’ inclusion criteria, outcomes measures, age and gender distributions of the included population, and authors. The two researchers will assess the presence of multiple publications of the same study within the review (KH, KMH).

## Data items

Information will be extracted from each included article by adapting the “Strengthening the Reporting of Observational studies in Epidemiology” (STROBE) checklist (9). The items are listed in the data extraction form (Appendix 2).

## Risk of bias in individual studies

With the absence of a defined bias assessment tool for observational studies and as there is no consensus on how to synthesise information about quality from a range of study designs within a systematic review (10), some items of STROBE checklist are used as quality indicators to assess the presence of potential bias in the reviewed studies (9).

The quality assessment will be done in conjunction with data extraction. In addition to the initial eligibility criteria, the minimum quality required is that the ADRs are defined according to WHO’s or a similar definition. If the authors use another definition close to WHO’s, the two reviewers will assess independently if it fits WHO’s definition and decide by consensus to include or not the study.

The assessment methods of causality and preventability will also be reported (number of assessors, causality/preventability instrument). A study will be included if the percentage of patients with PADRs or the preventability is clearly reported with the possibility to calculate the variance of the measured outcome (using the standard deviation, standard error or confidence interval), and that even if the study design is not clearly reported in the article. Because some items included may require a degree of subjective judgment, the two reviewers will gauge independently the presence of bias in reviewed studies and will document their conclusion. Any disagreement in the bias assessment will be discussed and resolved by consensus between the two reviewers.

When possible, risks of bias are identified. Further, sensitivity analyses will be realised (see next section).

We will also assess the possibility of publication bias by evaluating funnel plots.

# Summary measures

The total number of healthcare visits and those due to PADRs will be used from each study for calculating the percentage of patients with PADRs. For preventability, the total number of ADRs and PADRs will be used from the studies.

For interventional studies, data of the control arm with baseline values will be used. For case-control studies, we will use case group data (people having the PADRs).

If a study investigates PADRs occurring in both outpatients and inpatients (e.g. those present at the time of hospitalisation and occurring during hospitalisation), these will be separated for the analysis and they will have the same source population.

# Data synthesis

All studies included in the review will be first described in a table according to their main characteristics and outcomes measures.

Similar studies in terms of settings and population age will be grouped together in order to explore the possibility to conduct a meta-analysis. Studies will be divided according to:

- Whether ADRs occurred in outpatients or inpatients:
  1. PADR(s) occurred in outpatients, e.g. was present during a primary or emergency care visit, or at the time of hospitalisation
  2. PADR(s) was present in inpatients during hospitalisation.
- Age groups: all ages, only non-elderly adults, only elderly.

Meta analyses of the percentage of patients with PADRs and the preventability of ADRs will be conducted separately for these groups (if possible considering the number of studies). Studies reporting PADRs among outpatients will be pooled together, but will also be investigated separately based on the unit of detection (primary care, emergency care, hospitalisation).

## Aggregation percentage of patients with PADRs and preventability estimates across studies

The percentage of patients with PADRs in individual studies will be calculated by dividing the reported number of healthcare visits with PADRs by the total number of healthcare visits. These percentages will be calculated only if the number of healthcare visits is interpretable in the original studies.

The preventability of ADRs will be calculated by dividing the number of PADRs by the total number of ADRs.

Summary measures for the percentage of patients with PADRs and for the preventability of ADRs will be calculated separately for 1) ADRs occurring in outpatient settings and, 2) ADRs present during hospitalisation.

To calculate the summary measures across studies, we will multiply each study’s measures by their weights (inverse variance), sum the weighted estimates across studies, and then divide them by the sum of weights. To meet the normal distribution assumption, individual studies’ estimates will be converted to logit, the analysis will be conducted on the logit and the final results will be converted back to non-logit for interpretation. For comparison, the analyses will also be calculated using direct estimates, without converting them into logit. If the results of the direct and logit methods do not differ, the direct method will be chosen. Confidence intervals (95%) for each summary measure will also be calculated.

The percentage of patients with PADRs and preventability estimates will be presented graphically in a forest plot which illustrates the effect estimates from individual studies and the overall summary estimate if results are pooled in a meta-analysis. It also gives a good visual summary of variation between-study results.

If individual studies are too dissimilar to calculate an overall summary estimate of effect, a forest plot that omits the summary value can be produced (10).

## Heterogeneity

To reduce heterogeneity, we will include only studies fitting WHO’s or similar definition of ADRs. In order to avoid additional heterogeneity between studies, we will also measure separately the percentage of inpatients and outpatients with PADRs and the preventability of ADRs for inpatients and outpatients. However, we are aware of the high probability of heterogeneity between studies. Thus, heterogeneity as a measure of consistency between studies will be assessed using Cochrane’s Q test of heterogeneity and I^2^ statistics. Heterogeneity is expected considering different study designs, populations and health systems. To correct for heterogeneity, we will use the random coefficient model to pool the estimates of the studies. Despite heterogeneity, a meta-analysis provides evidence of the overall percentage of patients with PADRs and the preventability of ADRs.

## Sub-group analysis

Subgroup analysis will be conducted to measure the percentage of patients with PADRs in outpatients and inpatients. Studies will be also divided according to the population age groups. If results can not be pooled because of a low power, we will describe these studies and discuss their results.

## Sensitivity analysis

We will compare the overall estimates for the percentage of patients with PADRs and the preventability of ADRs with estimates obtained after iterations using N-1 studies (N is the total number of individual studies included in the subgroup). We will remove one study result each time and calculate the overall estimate. Then, we will replace that result, remove another and repeat the process until all studies whose estimates differ 20% or more from the overall estimate are omitted from each analysis. The aim is to measure the effect on individual studies estimations on the overall estimate. It is particularly useful in measuring the effect of individual studies on the overall estimate if we suspect that the results of one study differ from the others, especially if we judge that risk of bias may be present.

Each analysis will also be conducted separately for studies with less than six months’ study period and with six months’ or longer study period, because studies with longer study periods may more likely include readmissions of the same patients.

**References**

1. Lazarou J, Pomeranz BH, Corey PN. Incidence of adverse drug reactions in hospitalized patients: A meta-analysis of prospective studies. *JAMA*. 1998 Apr 15;279(15):1200-5.

2. Jonsson AK, Hakkarainen KM, Spigset O, Druid H, Hiselius A, Hagg S. Preventable drug related mortality in a swedish population. *Pharmacoepidemiol* *Drug Saf*. 2010 Feb;19(2):211-5.

3. Field TS, Gilman BH, Subramanian S, Fuller JC, Bates DW, Gurwitz JH. The costs associated with adverse drug events among older adults in the ambulatory setting. *Med Care.* 2005 Dec;43(12):1171-6.

4. WHO, Medicines: Safety of medicines- adverse drug reactions.; Available from: [*http://www.who.int/mediacentre/factsheets/fs293/en/*](http://www.who.int/mediacentre/factsheets/fs293/en/), accessed :12/12/2010.

5. Goettler M, Schneeweiss S, Hasford J. Adverse drug reaction monitoring--cost and benefit considerations. part II: Cost and preventability of adverse drug reactions leading to hospital admission. *Pharmacoepidemiol Drug Saf.* 1997 Oct;6 Suppl 3:S79-90.

6. Beijer HJ, de Blaey CJ. Hospitalisations caused by adverse drug reactions (ADR): A meta-analysis of observational studies. *Pharm World Sci*. 2002 Apr;24(2):46-54.

7. Kongkaew C, Noyce PR, Ashcroft DM. Hospital admissions associated with adverse drug reactions: A systematic review of prospective observational studies. *Ann Pharmacother*. 2008 Jul;42(7):1017-25.

8. Liberati A, Altman DG, Tetzlaff J, Mulrow C, Gotzsche PC, Ioannidis JP, et al. The PRISMA statement for reporting systematic reviews and meta-analyses of studies that evaluate health care interventions: Explanation and elaboration. *J Clin Epidemiol*. 2009 Oct;62(10):e1-34.

9. Von Elm E, Altman DG, Egger M, Pocock SJ, Gotzsche PC, Vandenbroucke JP, et al. The strengthening the reporting of observational studies in epidemiology (STROBE) statement: Guidelines for reporting observational studies. *J Clin Epidemiol*. 2008 Apr;61(4):344-9.

10. In: *Systematic reviews*. ed: Centre for Reviews and Dissemination, University of York, Available from:(<http://www.york.ac.uk/inst/crd/pdf/Systematic_Reviews.pdf>)

**Appendix 1.** Search strategy used in the database search of MEDLINE, Excerpta Medica Database (EMBASE), the Cochrane database of systematic reviews, Cumulative Index to Nursing & Allied Health Literature (CINAHL), International Pharmaceutical Abstract (IPA), PsycINFO and Web of Science

**#1** Abstract, title and index term search with exact phrase:

adverse drug effect

adverse drug effects

adverse drug event

adverse drug events

adverse drug reaction*

adverse drug reactions*

OR

drug induced disease

drug induced diseases

drug related morbidity

drug related problem

drug related problems

medication error

medication errors

*used only for title and abstract in EMBASE

**#2** Abstract, title and index term search with exact phrase:

avoidable

avoidability

preventability

OR

preventable

prevention

preventive

**#3** #1 AND #2

| **Appendix 2.** Data extraction form | | | | | | | |
| --- | --- | --- | --- | --- | --- | --- | --- |
| **Use of the item** | **Data to collect** | **Items** | | | | | |
| **R, Q** | **Frequency of ADRs an outcome measure** |  Yes   No → **Exclude** | | | | | |
| **R, Q** | **Frequency of PADRs or preventability of ADRs an outcome measure** |  Yes, frequency of PADRs   Yes, preventability of ADRs   No, neither → **Exclude** | | | | | |
| **MP, R** | **Year /period of the study** |  | | | | | |
| **R** | **Duration of data collection** |  | | | | | |
| **MP, R** | **First author** |  | | | | | |
| **MP, R** | **Study country** |  | | | | | |
| **R** | **Study design/**  **type of study** |  Observational | | | | | |
|  |  |  Retrospective | | | | | |
|  |  |  Prospective | | | | | |
|  |  |  Cross-sectional | | | | | |
|  |  |  Intervention | | | | | |
|  |  |  Not defined | | | | | |
| **R** | **Data source** |  Case records | | | | | |
|  |  |  Interview | | | | | |
|  |  |  Reports | | | | | |
|  |  |  Survey | | | | | |
|  |  |  Direct observation | | | | | |
|  |  |  Other: | | | | | |
| **MP, R** | **Sampling frame** |  | | | | | |
|  |  |  Not reported | | | | | |
| **R** | **Setting** |  Inpatient setting | | | | | |
|  |  |  Outpatient setting | | | | | |
|  |  |  Other setting: | | | | | |
|  |  |  No settings restrictions | | | | | |
|  |  |  Not reported → **Exclude** | | | | | |
| **MP, R** | **Participants characteristics** | **Age** | Mean(y): | | Median(y): | | |
|  |  |  | Range: | | | | |
|  |  |  | Reported in general terms: | |  Not reported | | |
|  |  | **Gender** | Male (%): | | Female (%): | | |
|  |  |  | Sex ratio: | | | | |
|  |  |  | Not reported | | | | |
| **MP,R,Q** | **ADR definition** |  Exactly according to WHO | | | | | |
|  |  | Other definition   Does not fit WHO definition→ **Exclude** | | | | | |
|  |  |  Not defined→ **Exclude** | | | | | |
| **R, Q** | **Number of assessors for causality/case** |  | | | | | |
| **R, Q** | **Assessment instrument for causality** |  | | | | | |
| **R, Q** | **Number of assessors for preventability/case** |  | | | | | |
| **R, Q** | **Assessment instrument for preventability** |  | | | | | |
| **MP,R,Q** | **Numerical values for percentage of patients with PADRs** | Incidence | | Prevalence | | Other frequency measure |  Not reported |
| **R, Q** | **Total number of persons and/or healthcare visits with ADR** |  | |  Not reported/ interpretable | |  All not reported → **Exclude from analysis of patients with PADRs** |  Data for neither patients with PADRs or preventability of ADRs presented → **Exclude** |
| **R, Q** | **Total number of persons and/or healthcare visits included** |  | |  Not reported/ interpretable | |  |  |
| **R, Q** | **Total number of persons and/or healthcare visits with PADR** |  | |  Not reported/ interpretable | |  |  |
| **R, Q** | **Preventability of ADRs** | Total number of ADRs:  Total number of PADRs:  (PADR/ADR)*100%: | | | |  <2 defined → **Exclude from ADRs’ preventability analysis** |  |

**Abbreviation**: **ADR:** Adverse Drug Reaction; **PADR**: Preventable Adverse Drug Reaction; **WHO**: World Health Organization; **R**: Results; **Q**: Quality evaluation of reviewed article**; MP**: Multiple Publication of the same study.
